# Developing Cardiac and Skeletal Muscle Share Fast-Skeletal Myosin Heavy Chain and Cardiac Troponin-I Expression

**DOI:** 10.1371/journal.pone.0040725

**Published:** 2012-07-10

**Authors:** Kelly C. Clause, Jason Tchao, Mary C. Powell, Li J. Liu, Johnny Huard, Bradley B. Keller, Kimimasa Tobita

**Affiliations:** 1 Cardiovascular Development Research Program, Children’s Hospital of Pittsburgh of University of Pittsburgh Medical Center, University of Pittsburgh, Pittsburgh, Pennsylvania, United States of America; 2 Department of Bioengineering, University of Pittsburgh, Pittsburgh, Pennsylvania, United States of America; 3 Department of Developmental Biology, University of Pittsburgh, Pittsburgh, Pennsylvania, United States of America; 4 Department of Orthopedic Surgery, University of Pittsburgh, Pittsburgh, Pennsylvania, United States of America; 5 McGowan Institutes for Regenerative Medicine, University of Pittsburgh, Pittsburgh, Pennsylvania, United States of America; 6 Department of Pediatrics, University of Louisville, Louisville, Kentucky, United States of America; Leiden University Medical Center, The Netherlands

## Abstract

Skeletal muscle derived stem cells (MDSCs) transplanted into injured myocardium can differentiate into fast skeletal muscle specific myosin heavy chain (sk-fMHC) and cardiac specific troponin-I (cTn-I) positive cells sustaining recipient myocardial function. We have recently found that MDSCs differentiate into a cardiomyocyte phenotype within a three-dimensional gel bioreactor. It is generally accepted that terminally differentiated myocardium or skeletal muscle only express cTn-I or sk-fMHC, respectively. Studies have shown the presence of non-cardiac muscle proteins in the developing myocardium or cardiac proteins in pathological skeletal muscle. In the current study, we tested the hypothesis that normal developing myocardium and skeletal muscle transiently share both sk-fMHC and cTn-I proteins. Immunohistochemistry, western blot, and RT-PCR analyses were carried out in embryonic day 13 (ED13) and 20 (ED20), neonatal day 0 (ND0) and 4 (ND4), postnatal day 10 (PND10), and 8 week-old adult female Lewis rat ventricular myocardium and gastrocnemius muscle. Confocal laser microscopy revealed that sk-fMHC was expressed as a typical striated muscle pattern within ED13 ventricular myocardium, and the striated sk-fMHC expression was lost by ND4 and became negative in adult myocardium. cTn-I was not expressed as a typical striated muscle pattern throughout the myocardium until PND10. Western blot and RT-PCR analyses revealed that gene and protein expression patterns of cardiac and skeletal muscle transcription factors and sk-fMHC within ventricular myocardium and skeletal muscle were similar at ED20, and the expression patterns became cardiac or skeletal muscle specific during postnatal development. These findings provide new insight into cardiac muscle development and highlight previously unknown common developmental features of cardiac and skeletal muscle.

## Introduction

Muscles are composed of different fiber types to fulfill various functional needs. Fiber types are categorized generally according to their specific myosin heavy chain (MHC) isoforms. In rats, there are four major isoforms of MHC, one slow type (type I/ß) and three fast types: IIa, IIx/IId, and IIb, which is equivalent to skeletal muscle specific fast myosin heavy chain (sk-fMHC). An individual muscle fiber can contain just one myosin isoform or mixtures of two or more different isoforms [1], [2], [3]. An additional MHC isoform, α, is present in the myocardium. Different MHC isoforms are expressed in both tissue and stage-specific manners, and much work has been done to show the relative change in the expression ratio of α-MHC: ß-MHC in the myocardium during development and with intervention. During the early myofibrillogenesis of nascent cardiomyocytes, non-cardiac MHC plays an important role in assembling sarcomere structure [4]. However, little investigation into the expression of skeletal muscle specific MHCs in the developing myocardium has been done.

Troponins are proteins that regulate the thin filament system in skeletal and cardiac muscle and form part of the contractile complex. Troponin I is encoded by 3 different genes and is expressed differentially in various types of tissue. However, cardiac troponin I (cTn-I) is uniquely expressed in the heart and is distinct from the fast and slow forms in skeletal muscle [5].

It has been widely accepted that terminally differentiated mature cardiac muscle does not express proteins that are specific to skeletal muscle. However, studies have shown that several skeletal muscle specific proteins, such as skeletal muscle specific troponins, are transiently present in the developing heart [6]. Similarly, “cardiac” and “skeletal” excitation-contraction coupling mechanisms co-exist in developing skeletal muscle with the “cardiac” type dominant in the early phases of myogenesis and the “skeletal” dominating in more mature muscle [7], [8]. These studies suggest the coexistence of many cardiac and skeletal muscle specific proteins and excitation-contraction coupling mechanisms within both developing tissue and cultured cells, particularly those that are considered to be immature. While the idea that skeletal and cardiac muscle share partially overlapping developmental profiles is not new, reports of expression of specific structural protein isoforms have varied across studies. For example, Fougerousse et al. concluded that the cardiac isoform of myosin binding protein C, cardiac MyBP-C, is strictly specific to the heart during murine and human development [9]. However, it is reported to be expressed in developing chick skeletal muscle [10], [11], [12]. Factors such as experimental conditions, differences across species, and developmental time points examined can lead to these differences in results. Moreover, while these phenotypic changes have been studied during early cardiac morphogenesis period, few have focused on the transition that occurs between late fetal and early postnatal life.

In our previous studies of murine or human skeletal muscle derived stem cell (MDSC) transplantation into infarcted mouse myocardium, we found that MDSC transplanted post-infarcted myocardium sustains its contractile function, preventing left ventricular chamber remodeling, and some transplanted donor MDSCs differentiate into both sk-fMHC and/or cTn-I positive muscle cells [13], [14]. We have recently shown MDSCs can differentiate into cells with an immature functioning cardiomyocyte phenotype within a three-dimensional engineered tissue construct [15]. However, MDSC derived cardiomyocytes generated *in vitro* are biochemically and functionally more similar to fetal rather than mature cardiomyocytes. While stem cell derived cardiomyocytes show promise, the sequence of events that lead to terminal cardiomyocyte differentiation and functional maturation is as of yet poorly understood [16]. The use of stem-cell derived cardiomyocytes for tissue engineering and regenerative medicine applications would clearly benefit from a better understanding of the biochemical and structural changes that lead to cardiac and skeletal muscle specification during natural development. However, it remains to be seen whether MDSC derived sk-fMHC positive cells are terminally differentiating skeletal muscle cells or potentially differentiating cardiomyocytes. In order to help address the question, we investigated the presence of sk-fMHC and cTn-I within the native developing myocardium and skeletal muscle in a rat animal model.

## Materials and Methods

The presence of sk-fMHC and cTn-I from embryonic day (ED) 13 and 20, neonatal day (ND) 0 and 4, postnatal day (PND) 10, and 8 week-old adult female Lewis rat ventricular myocardium and skeletal muscle (hind limb in ED13, ED20, ND0 and ND4, and gastrocnemius and soleus muscles in PND10 and adult) was assessed using immunohistochemistry, western blot, and RT-PCR. Our research protocol followed the National Institutes of Health (NIH) guidelines for animal care and was approved by the University of Pittsburgh’s Institutional Animal Care and Use Committee.

**Figure 1:**
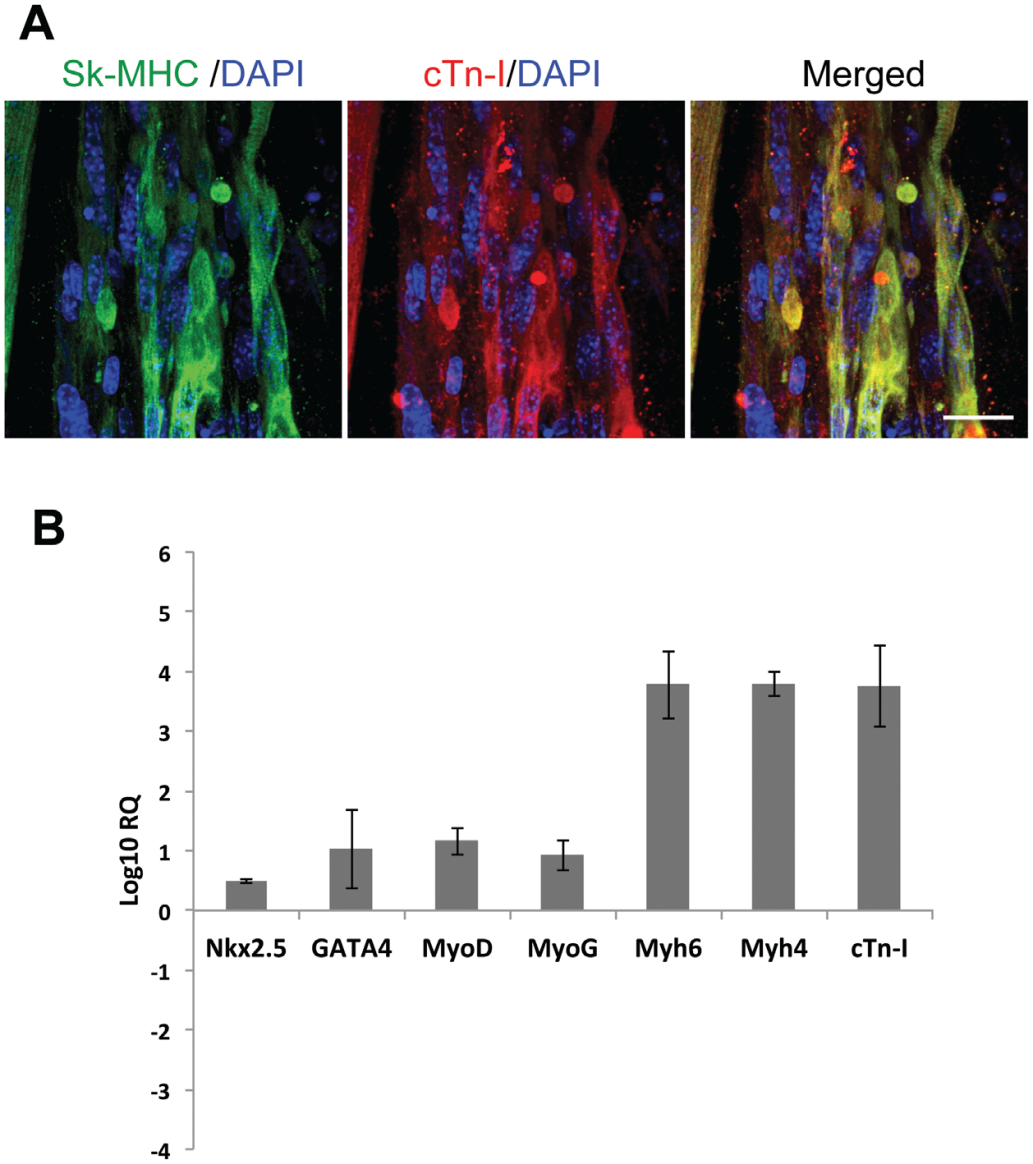
sk-fMHC (green color) and cardiac troponin-I (red color) expression (Panel A) and gene expression (Panel B) of skeletal muscle derived stem cell 3D collagen gel bioreactor (MDSC-3DGB). MDSC-3DGB showed co-localized expression of sk-fMHC and cardiac troponin I. Transcription factor and structural gene expression was increased compared to 2D undifferentiated MDSCs. Scale in panel A indicates 20 µm.

### Construction of 3-dimensional Collagen Gel Bioreactor from Rat Skeletal Muscle Derived Stem Cells (MDSCs)

We isolated skeletal muscle derived stem cells (MDSCs) from 3 post-natal day 10 Lewis rat gastrocnemius muscle using a preplate technique [15]. Isolated MDSCs were expanded on a rat-tail collagen type-1 (Invitrogen, Carlsbad, CA, USA) coated flask (T-75, Fisher Scientific, Pittsburgh, PA) and a 3-dimensional collagen gel bioreactor was constructed using a mixture of MDSCs and rat tail collagen type-I (Invitrogen) with Matrigel (BD bioscience, San Jose, CA, USA), and a Flexcell 4000TT system (Flexcell International, Inc. Hillsborough, NC, USA) [15]. The constructed collagen gel tissue was cultured for 9 days and the tissue was harvested for histological assessment and the gene expression [15].

**Figure 2:**
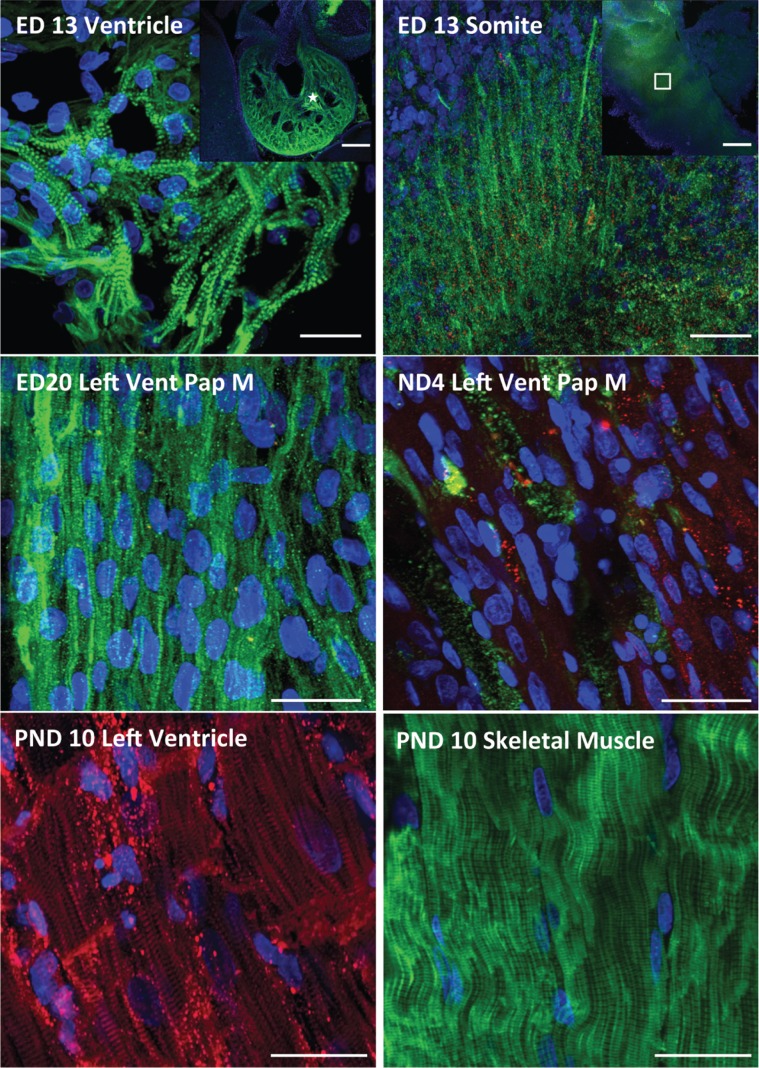
sk-fMHC (green color) and cardiac troponin-I (red color) expression within developing ventricular myocardium and skeletal muscle. The skeletal muscle fast myosin heavy chain (sk-fMHC) and cardiac troponin-I (cTn-I) expression within the embryonic day (ED13) heart (ED13 Ventricle insert, scale indicates 500 µm). The sk-fMHC was expressed as a typical striated muscle pattern in the developing ventricle and cTn-I expression was near background level at ED13. Somites also express sk-fMHC as a fiber structure and cTn-I was also expressed very weakly similar to the heart (ED13 somite panel). Scales in ED13 ventricle and somite panels indicate 20 µm. At ED20, both heart muscle (ED20 Left Vent Pap M) and skeletal muscle (not shown) express sk-fMHC and cTn-I as a striated muscle pattern. Scales in ED20 and ND4 Vent Pap M panels indicate 20 µm. In the heart, sk-fMHC expression is significantly decreased, and cTn-I expression was increased with striation pattern after neonate day 4, and skeletal muscle significantly decreases its cTn-I expression after neonate day 4 (data not shown). At postnatal day 10 (PND10), left ventricular myocardium does not express sk-fMHC and cTn-I displayed a typical striation pattern (PND10 Left Ventricle panel). Conversely, gastrocnemius muscle expressed sk-fMHC as a typical striation pattern and cTn-I was negative (PND10 skeletal muscle panel). Scales in PND10 panels indicate 20 µm.

### Immunohistochemical Staining

Heart and skeletal muscle samples, and MDSC-collagen gel constructs (N = 4) were fixed with 4% paraformaldehyde/PBS for 15 minutes (EDs 13 and 20, N = 4 in each developmental stage) to 12 hours (ND0 to adult) and embedded in 13% polyacrylamide gel. The 100 (ND4 and adult) to 150 µm (ED13, ED20, ND0) thick sections were made using a vibratory microtome (Vibratome-1000, Vibrotome.com) [17] and stained for mouse monoclonal sk-fMHC (Sigma MY32, St. Louis, MO, USA, 1∶200 dilution) and mouse monoclonal cardiac specific Troponin-I [cTn-I, Abcam (19C7), Cambridge, MA, USA, 1∶100 dilution] primary antibodies and Alexa Fluor 488 IgG_2b_ or Alexa Fluor 594 IgG_1_ goat anti-mouse secondary antibodies (Invitrogen, Carlsbad, CA, USA, 1∶200 final concentration). The stained sections were optically scanned with a Z-stack imaging protocol (from 20 to 30 optical sectioning/filed) by using a standard laser scanning confocal microscope (FV1000, Olympus, Tokyo, Japan) [17]. Imaging fields (at least 5 different fields/sample) were randomly selected and the acquired image stack was used to make a 3D projection image using Image-J software (NIH).

**Figure 3:**
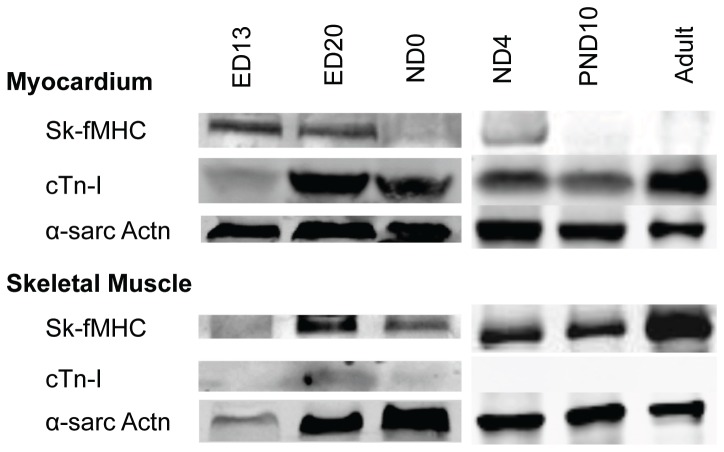
Western blot analysis of developing myocardium and skeletal muscle. **Lane 1**: ED13; **Lane 2**: ED20; **Lane 3**: ND0; **Lane 4**: ND4; **Lane 5**: PND10; **Lane 6**: Adult (8 week-old). **Top panel**: Left ventricular myocardium, **Bottom panel**: hind limbs at ED13, ED20, ND0, and ND4, and gastrocnemius muscle in PND10 and adult. At ED13 and ED20, left ventricular myocardium expressed sk-fMHC and cTn-I expression was very weak while skeletal muscle expressed sk-fMHC. During development, sk-fMHC expression in the left ventricular myocardium decreased and increased in skeletal muscle, whereas cTn-I is expressed in the left ventricular myocardium and was negative in skeletal muscle. α-sarcomeric actinin (α-sarcActn) was used to adjust the total protein loading for the electrophoresis.

### SDS-PAGE and Immunoblotting

Freshly frozen heart, skeletal muscle, and MDSC-collagen gel constructs (n = 3) samples were used. Protein was extracted from pooled samples, and immunoblotting was carried out using routine protocols. Each lane contained 20 µg of total protein. Mouse monoclonal α-sarcomeric actinin (Sigma, EA53, 1∶500 dilution), mouse monoclonal cardiac troponin-I (Abcam, 1∶100 dilution), and mouse monoclonal sk-fMHC (Sigma MY32, 1∶500 dilution) were visualized with IR-Dye 800 donkey anti-mouse secondary antibody (IgG_1_ and IgG_2b_, Rockland Immunochemicals, Gilbertsville, PA, USA, 1∶10,000 dilution). All proteins were visualized using an infrared western blot imaging system (Odyssey, LI-COR Biosciences Lincoln, NE, USA). Immunoblots were performed in either quintuplicate or triplicate [ED 13 (n = 30), ED 20 (n = 5), ND 0 (n = 5); n = 3 for ND4 (n = 3), PND 10 (n = 3), and adult rats (n = 3)] [15].

**Figure 4:**
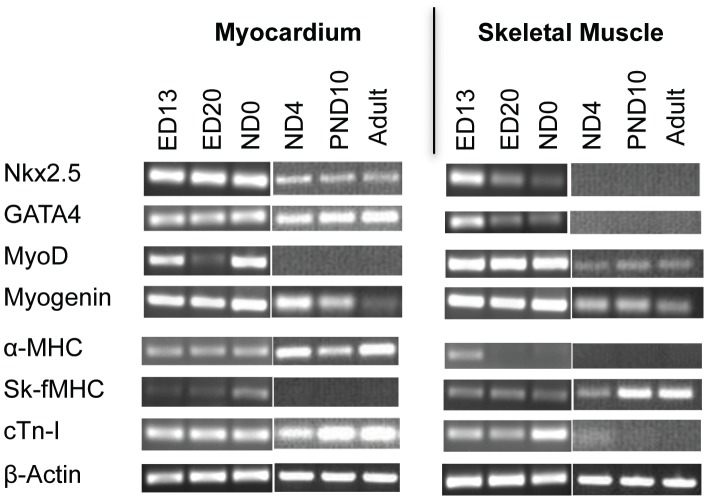
RT-PCR analysis of cardiac and skeletal muscle transcription factors, sk-fMHC, cTn-I mRNA expression. **Lane 1**: ED13 ventricle, **Lane 2**: ED20 ventricle; **Lane 3**: ND0 ventricle; **Lane 4**: ND4 ventricle; **Lane 5**: PND10 ventricle; **Lane 6**: Adult ventricle; **Lane 7**: ED13 hind limbs; **Lane 8**: ED20 hind limbs, **Lane 9**: ND0 hind limbs; **Lane 10**: ND4 hind-limbs; **Lane 11**: PND10 gastrocnemius muscle; **Lane 12**: Adult gastrocnemius muscle. We note that ED20 skeletal muscle (hind limbs) mRNA expression patterns are similar to ED20 ventricular myocardium.

### RT-PCR and Real-time RT-PCR

Freshly frozen pooled ventricular myocardium, skeletal muscle samples [ED13 (n = 10), ED20 and ND0 (n = 5), ND4, PND10, and adult (n = 3)], and MDSC-collagen gel constructs (n = 3) were used. Total RNA was prepared using Trizol solution (Invitrogen) and treated with TURBO DNA-free kit (Ambion, Austin, TX, USA). Primers, whose target genes were, Nkx2.5, GATA4, MyoD, Myogenin, sk-fMHC (MYH4), α-MHC (MYH7), cardiac troponin-I (Tnn3), were obtained from Qiagen Quanti-Tect Primer Assay (Qiagen, Valencia, CA). One-step reverse transcription (RT) was performed with a total amount of 1 µg RNA in a total volume of 25 µL that used MuLy (Roches, Pleasanton, CA, USA). The following program was used for RT: 42°C 15 minutes, 99°C 5 minutes, 5°C 5 minutes, for 1 cycle. The produced cDNA (1 µL) was used for RT-PCR (94°C 2 minutes, 95°C 50 seconds, 58°C 30 seconds, 72°C 1 minute, for 30 cycles followed by 72°C 7 minutes extension) or real-time RT-PCR (50°C for 2 minutes, 95°C for 10 minutes, 95°C for 15 seconds, 60°C for 1 minute, 95°C for 15 minutes, 60°C for 15 minutes for 40 cycles). For normalization of real-time PCR results, β-actin was used as an internal control. All PCR products were confirmed by University of Pittsburgh DNA Sequence Core Facilities, performed by Eppendorf Mastercycles. All real-time PCR assays were completed in triplicate (n = 3 in each developmental stage). Real-time PCR samples were processed using Applied Biosystems HT7900 system.

**Table 1:** Cycle to threshold (Ct) Values of cardiac and skeletal muscle transcription factor and structural muscle protein gene expression of Embryonic day 13 (ED13) ventricular myocardium and skeletal muscle (limbs).

	Beta Actin	Nkx2.5	GATA4	MyoD	Myogenin	MYH6	MYH4	cTn-I
**Skeletal Muscle**	14.5±0.2	25.8±0.1	28.6±0.4	22.5±0.5	20.8±0.0	23.4±0.6	28.9±0.3	22.9±0.0
**Heart**	15.8±0.1	21.1±0.2	24.5±0.2	32.5±0.2	27.9±0.1	34.1±1.6	36.1±0.9	16.3±0.1

Although expression levels vary between both types of tissue, their transcriptional and structural gene profiles overlap.

## Results

Differentiating MDSCs in a 3-dimensional collagen/extracellular matrix gel construct displayed spontaneous beating activity from culture day 5 and formed a muscle tissue by culture day 7 similarly to our previous study [15]. Confocal microscopy showed that the differentiating cardiomyocyte-like cells co-expressed skeletal muscle specific fast myosin heavy chain (sk-fMHC) and cardiac specific troponin-I (cTn-I) as a typical striated muscle pattern (**Figure 1** **, panel A**). Real time RT-PCR showed that both cardiac and skeletal muscle transcription factors, cardiac sk-fMHC, cardiac α-myosin heavy chain, and cTn-I genes are up-regulated compared to undifferentiated MDSC (**Figure 1** **, panel B**).

**Figure 5:**
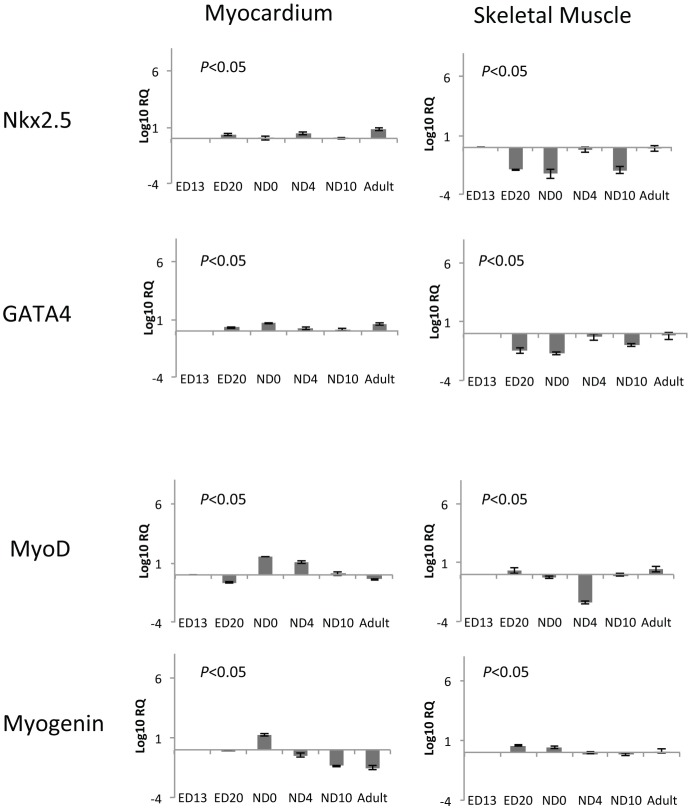
Changes in mRNA levels of cardiac and skeletal muscle specific transcription factors within ventricular myocardium and skeletal muscle. Data are expressed as average ± SD. Cardiac transcription factors, Nkx2.5 and GATA4, and skeletal muscle transcription factor MyoD expression did not change in ventricular myocardium in adulthood, whereas myogenin expression was significantly decreased in ventricular myocardium (*P*<0.05, ANOVA). Nkx2.5 and GATA4 were significantly decreased during development, and MyoD and myogenin were significantly increased in skeletal muscle during the development (*P*<0.05). Log_10_RQ: Relative Quantification of mRNA level compared to the mRNA level in ED13. The ratio is expressed as logarithm with base value of 10 (Log_10_).

In the native rat heart, sk-fMHC was expressed as a typical striated muscle pattern throughout the entire ventricular myocardium at ED13, and cTn-I was very weakly expressed (close to background) (**Figure 2** **)**. Within ED13 somite, sk-fMHC was expressed as a striated thin fiber-like pattern, while cTn-I was diffusely expressed. At ED20 and ND0, striated sk-fMHC expression was restricted to the ventricular papillary muscle and losing striated muscle pattern in the myocardial wall (**Figure 2**). cTn-I was very weakly expressed without striation in the majority of the myocardial wall and some striated cTn-I expression pattern was identified in the myocardium close to LV apex (Data not shown). At ND4, sk-fMHC was still expressed in the LV myocardium (**Figure 2**). However, striated pattern was completely lost. cTn-I expression increased. The PND10 LV myocardium did not express sk-fMHC, whereas cTn-I was expressed with a typical striated pattern. Conversely, PND10 skeletal muscle expressed sk-fMHC as a typical striated muscle pattern and did not express cTn-I (**Figure 2**).

Western blot analysis indicated that in the myocardium, sk-fMHC was expressed in decreasing amounts until PND10 (juvenile) and was not expressed in the adult myocardium (8 week-old), whereas cTn-I was expressed in increasing amounts at all developmental stages. In skeletal muscle, sk-fMHC was expressed at all developmental stages, while cTn-I was expressed very weakly at ED13 and the expression increased at ED20, decreased at ND0, and became negative after ND4 (**Figure 3**).

**Figure 6:**
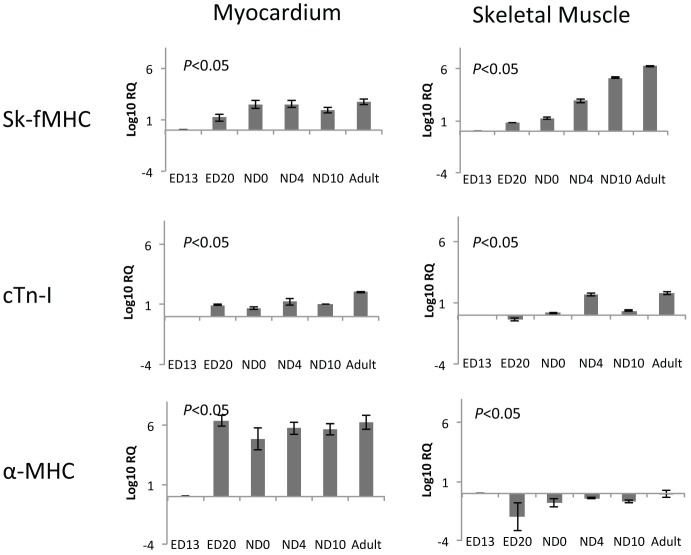
Changes in mRNA levels of sk-fMHC, cTn-I, and cardiac α-MHC within ventricular myocardium and skeletal muscle. Data are expressed as average ± SD. The sk-fMHC mRNA level was significantly decreased and cTn-I and α-MHC levels were significantly increased in ventricular myocardium (*P*<0.05, ANOVA). Conversely, sk-fMHC mRNA level was significantly increased and cTn-I and α-MHC were significantly decreased in skeletal muscle. Log_10_RQ: Relative Quantification of mRNA level compared to the value in ED13. The ratio is expressed as logarithm with base value of 10 (Log_10_).

RT-PCR analysis (30 PCR cycles, **Figure 4**) showed that in ventricular myocardium, cardiac transcription factors, Nkx2.5 and GATA4, were expressed through all developmental stages and were also expressed in gradually decreasing levels in skeletal muscle. Skeletal muscle transcription factor MyoD was detected at 30 PCR cycles in skeletal muscle at all developmental stages and in ventricular myocardium until ND0. Another major skeletal muscle transcription factor, myogenin, was expressed in both ventricular myocardium and skeletal muscle in all developmental stages. The sk-fMHC mRNA was expressed in ND0 myocardium, and the expression was significantly decreased at ND4. It was then negative in PND10 and adult ventricular myocardium. The sk-fMHC mRNA was expressed at all developmental stages in the skeletal muscle. The cardiac specific α-myosin heavy chain (α-MHC) was present in the ventricular myocardium at all developmental stages and ED13 skeletal muscle. Conversely, postnatal and adult skeletal muscle did not express α-MHC. cTn-I mRNA was expressed in ventricular myocardium in all developmental stages and ED13, ED20 and ND0 skeletal muscle, and became negative in PND10 and adult skeletal muscle.

We semi-quantified the changes in mRNA levels of the genes described above by real-time RT-PCR. Table 1 shows cycle to threshold (Ct) values of cardiac and skeletal muscle transcription factors, structural muscle protein, and β-actin mRNA of embryonic day 13 ventricular myocardium and skeletal muscle (limb). Although the Ct value of each transcription factor and structural protein mRNA varied, these gene expression patterns overlapped between both types of muscle tissues. During development, cardiac transcription factor, Nkx2.5 and GATA4, expression levels in postnatal ventricular myocardium remained at the same levels as ED13. The mRNA level of the skeletal muscle transcription factor, MyoD in the ventricular myocardium was elevated at ND0 and ND4 but remained the same as ED13 in postnatal stages, whereas the mRNA level of another skeletal muscle transcription factor, myogenin, was significantly decreased postnatally. In developing skeletal muscle, Nkx2.5 and GATA4 expression levels were significantly decreased, while MyoD and myogenin expression levels were transiently decreased at ND4 and were maintained at the same levels during the development (**Figure 5**). The sk-fMHC level in the myocardium increased from ED13 to ED20 and maintained a steady level thereafter, while it continuously increased in skeletal muscle. Conversely, cTn-I and α-MHC levels were significantly increased in postnatal ventricular myocardium. In skeletal muscle, cTn-I was increased at ND4 and adult, while α-MHC was decreased at ED20 and thereafter it was unchanged in postnatal development (**Figure 6**).

## Discussion

In the current study, we found that skeletal muscle specific fast myosin heavy chain (sk-fMHC) is transiently expressed in the developing immature myocardium, and cardiac troponin I (cTn-I) is transiently expressed in developing skeletal muscle. A number of previous studies have looked at an assortment of myosin and troponin expression patterns in both the cardiac and skeletal muscle. α-MHC was found in both normal adult diaphragm and stimulated fast twitch muscles in the rabbit, human masseter and extraocular muscles, as well as the bag fibers of human, rat, and cat muscle spindles [18], [19]. From the opposite perspective, anti-anterior latissimus dorsi myosin antibodies showed specific reactivity with the cells of the conduction system in the rabbit heart [20]. Similarly, there is abundant evidence that myosin expression undergoes transitions during the development of both skeletal and cardiac muscle in a number of species. Sweeney et al. found that the earliest MHC detectable in both forms of striated muscle in chick embryos (cardiac and skeletal) was indistinguishable from cardiac ventricular myosin expressed in the adult heart but immunologically distinct from myosins expressed in later embryonic and adult skeletal muscle. This suggests that the initial developmental program for myosin expression in differentiating skeletal muscle may be related to that for cardiac ventricular myosin [21]. In a similar study, cardiac ventricular MHC was found in developing as well as regenerating anterior latissimus dorsi avian skeletal muscle [22]. In the case of cardiac muscle though, it was unclear whether the developmental program involved the co-expression of skeletal as well as cardiac isomyosins. In the current study, we found that skeletal muscle specific fast MHC was in fact transiently expressed during the initial developmental program of the developing fetal myocardium.

Cardiac troponin-I and -T are normally specific to the adult cardiac muscle. However, cTn-T isoforms have been shown to be expressed in human skeletal muscle in individuals suffering from Duchenne muscular dystrophy, polymyositis, and end stage renal disease [5] and in developing and regenerating rat skeletal muscle [23]. Fredericks et al. showed that cTn-I and cTn-T are expressed in adult rat skeletal muscle in response to denervation, but the protein expression levels were several thousand times lower than in the heart [24]. Messner et al. showed that mRNA from cTn-T and cTn-I are expressed in skeletal muscle in patients with Duchenne muscular dystrophy and several other myopathies [25]. In the present study, we found that cTn-I was transiently expressed during the early development of skeletal muscle. To our knowledge, this is the first comprehensive study of the time course expression of cTn-I during the normal development of skeletal muscle. Sutherland et al. examined cTn-I mRNA expression in the developing rat hindlimb by Northern blot but did not detect any cTn-I in skeletal muscle [26]. This may be due to the low sensitivity of Northern blot relative to RT-PCR. We have shown that cTn-I is expressed in early developing skeletal muscle at both the gene and protein level and visually shown changes in its structural organization.

As development of the myocardium progressed, sk-fMHC became negative, and the myocardium then expressed cTn-I. Conversely, as development of skeletal muscle progressed, cTn-I became negative, and the skeletal muscle then expressed sk-fMHC. Fast skeletal muscle is more prone to fatigue, utilizes ATP quickly, and uses less oxygen, whereas cardiac muscle is made to resist fatigue and generally needs more oxygen. Thus, from a physiological standpoint, the benefit of sk-fMHC in the immature developing fetal myocardium may be shorter and quicker contraction for quicker blood ejection throughout the system, which is necessary for supporting rapid fetal growth prior to the establishment of coronary artery circulation, cardiac autonomic, and conduction systems. As the myocardium matures, the contraction pattern shifts towards less rapid but continuous contraction under aerobic conditions, at which point the coronary circulation and conduction as well as cardiac autonomic system are established, and sk-fMHC expression decreases. Continued expression of sk-fMHC would be detrimental because if fast muscle tissue were to persist in the postnatal and adult myocardium, it would fatigue much faster, potentially increasing the risk of cellular acidosis. A recent study by Rutland and colleagues reported the expression of embryonic myosin heavy chain (eMYH), a fast skeletal specific isoform, in the developing chick heart. Its functional homologue in humans is believed to be Myh3 [27]. While they did not detect the presence of other skeletal myosin heavy chain genes in human fetal and adult hearts, we speculate that sk-fMHC may serve a similar role in rats as Myh3 in humans during heart development. Moreover, while their study focused on changes that occur during early development, we place a specific emphasis on the events that occur during the transition from late fetal to early postnatal life.

One potential limitation of the current study is the use of the Sigma MY32 antibody and the lack of a more specific commercially available antibody. Sigma reports that it stains the fast (type II) and neonatal myosin molecules found in skeletal muscle. It is possible that MY32 may cross react with the embryonic and neonatal myosin heavy chain isoforms. The reactivity of this antibody with the embryonic and neonatal forms has not been fully characterized. However, Lyons et al. have reported that the embryonic and neonatal forms are not expressed in the developing mouse embryo heart [28], which would indicate that sk-fMHC is the only fast skeletal muscle MHC expressed in the developing heart in rats.

The N-terminal extension of cTn-I contains two phosphorylatable serines (Ser-23 and Ser-24), which are targets of protein kinase A and protein kinase C and are not present in the fast and slow skeletal isoforms of troponin I [29]. These additional sites confer a greater degree of regulation of Calcium sensitivity and contractility [29], [30]. Thus, the presence of the cardiac isoform in developing skeletal muscle might provide an additional level of control of muscle activity prior to the full development of the nervous system, which would increase energy efficiency in the metabolically active developing fetus.

Real-time RT-PCR analysis of gene expression in the developing heart and skeletal muscle supports the idea of developmental commonality between both types of tissue. Cardiac specific genes such as Nkx2.5, GATA4, a-MHC, and cTn-I are expressed in developing skeletal muscle. Expression levels of certain genes within the heart and skeletal muscle change over time, while others, notably Nkx2.5 and GATA4 within the myocardium, remain relatively constant over time. Interestingly, sk-fMHC mRNA continues to be expressed at low levels in adult myocardium, and cTn-I mRNA continues to be expressed at low levels in adult skeletal muscle, despite the absence of protein expression. It is possible that these genes are post-transcriptionally suppressed in adulthood, but the continued expression at the transcript level provides a faster means of response in response to external events such as injury such as myocardial infarction, which leads to recapitulation of the fetal gene program. Sassoon et al. reported that MyoD and myogenin were detected by in-situ hybridization in embryonic skeletal muscle but not heart [31]. However, they only specify that they examined expression in the heart at embryonic day 7.5 during heart tube formation. They do not specify the range of time points that they examined. MyoD and myogenin may only be expressed in the heart during later development, as our results indicate. Di Lisi et al. reported that GATA4 is expressed in C2C12 muscle cells at the transcript level but not at the protein level [32]. However, they used C2C12 cells terminally differentiated into myotubes in vitro under conventional 2D culture for their analysis, which does not accurately represent in vivo development. Overall, our results suggest a shared developmental program between skeletal and cardiac muscle, which is lost as they mature into distinct tissue types. We speculate that skeletal muscle development (maturation) occurs at later development compared to the cardiac muscle, and the skeletal muscle differentiation temporarily requires the cardiac muscle phenotype (cyclic twitch) to acquire the function towards highly controlled contraction and relaxation under the regulation of the nervous system, which may be supported by the previous report from Fredericks et al. [24].

Consistent with our pervious study, we showed that MDSCs isolated from juvenile rat skeletal muscle differentiate into cardiomyocyte like cells in a 3D collagen gel bioreactor (MDSC-3DGB) [15]. With respect to the current work, the MDSC-3DGB appeared to expresses cTn-I at higher levels than ED20 skeletal muscle but lower than ED20 myocardium. The findings presented here support the idea that MDSCs have the capacity to differentiate into a more cardiac phenotype in-vitro. Fate decisions are ultimately influenced by biochemical and biomechanical signals from the environment. Providing the appropriate environment for stem cell differentiation remains a topic of ongoing research.

In conclusion, the current study provides new evidence that skeletal muscle specific fast myosin heavy chain is transiently expressed in the developing immature myocardium, and cardiac troponin I is transiently expressed in developing skeletal muscle. These findings shed light on a previously unknown aspect of muscle development. Further studies are necessary to examine the role of these contractile proteins during development and how they are regulated.
